# Evidence for Reductions in Fungal Richness and Beta Diversity Dispersion Associated With Impervious Surfaces in California

**DOI:** 10.1002/ece3.74448

**Published:** 2026-09-29

**Authors:** Ariel Levi Simons, Christopher Timlin‐Broussard, Avicka Willis, Jerelyn Lee

**Affiliations:** ^1^ West Los Angeles College Culver City California USA; ^2^ Los Angeles City College Los Angeles California USA; ^3^ Santa Monica College Santa Monica California USA

**Keywords:** California, community science, ecological filtering, fungal diversity, fungi, urbanization

## Abstract

A large proportion of nutrient cycling and ecosystem services are supported by the activities of fungal communities. Though these communities have been comparatively understudied compared to more charismatic groups, there is a growing body of evidence that the structure of these communities is also impacted by urbanization. To investigate diversity patterns, we analyzed fungal occurrences recorded in California taken between 2015 and 2024. We investigated their differences in both richness and turnover by comparing communities sampled along a gradient of percent impervious coverage as a proxy for urbanization. We found evidence for a reduction in saprotrophic fungal richness for assemblages sampled from areas with higher levels of impervious coverage. We also found evidence that saprotrophic and symbiotrophic fungal assemblages tend to have reductions in beta diversity dispersion associated with a rise in impervious cover, potentially indicating an increase in biotic homogenization in more urbanized areas. In this study, we have demonstrated the potential use of processing large amounts of community science data to better understand the ecological responses of fungal communities against anthropogenic stresses.

## Graphical abstract

Using public fungal occurrence data archived on the Global Biodiversity information facility, and impervious land cover maps from the National Land Cover Database, we were able to demonstrate that impervious cover has a significant influence on the diversity on fungal assemblages. These effects include a reduction in saprotrophic fungal richness, and a reduction in beta diversity dispersion associated for saprotrophic and symbiotrophic fungal assemblages, associated with areas with greater levels of impervious coverage.

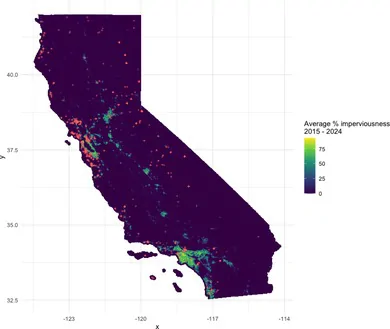

## 1 Introduction

Humanity has recently become a predominantly urban species, with the geographic scope of urban land cover expected to continue its rapid expansion over the coming century (McDonald 2008; Sun et al. 2020). Urbanization has been found to significantly shape ecological conditions ranging from local climate (Zhang et al. 2021) to patterns of biodiversity (Sidemo‐Holm et al. 2022). These landscape modifications, and their ecological impacts, are significant enough that urban areas can be considered a distinct class of biomes (Fleming and Bateman 2018; Teixeira and Fernandes 2020), containing their own unique ecosystems (Avolio et al. 2020). This has driven interest in studying urban areas as their own unique type of ecosystem (Montero 2020; Uchida et al. 2021).

The rise in the scale and intensity of anthropogenic stressors associated with urbanization makes the need for tracking its effects on biodiversity more pressing (Xu et al. 2021). This need for monitoring biodiversity covers everything ranging from establishing baselines (Linares 2022), monitoring spatiotemporal patterns (Magurran et al. 2010), determining extinction risks (Raimondo et al. 2023), and identifying predictors of community composition (Stupariu et al. 2022).

With traditionally understudied groups, in particular fungi, there are significant gaps in our monitoring efforts (Hochkirch et al. 2021; Niskanen et al. 2023). To help address these gaps we can make use of community science data. Community science, the collection or analysis of data by non‐professional scientists, has shown promise in enabling the collection of sets of species observations on a far larger scale than from individual research projects (McCaffrey 2005; Silvertown 2009; Kobori et al. 2016; Ballard et al. 2017; Spear et al. 2017). Such efforts have proliferated in the mycological community in recent years (Irga et al. 2020; Sheehan et al. 2021), generating large amounts of fungal data for biodiversity analyses (Hao et al. 2020; Wüest et al. 2020; Shumskaya et al. 2023; Yu 2023; Haelewaters et al. 2024) which are publicly available on the Global Biodiversity Information Facility (GBIF) archive (Steinke et al. 2025).

This study investigates the role of urbanization in shaping patterns of biodiversity for fungal communities in California, as archived in GBIF. We focus on California as it covers a diverse range of environmental conditions and biological communities (Myers et al. 2000), including biodiversity hotspots (Calsbeek et al. 2003). Additionally, California is of particular interest in studying the effects of urbanization on ecosystems as it is heavily urbanized (McDonald et al. 2013; Gillespie et al. 2018), and recently so (Harrison et al. 2024).

Fungal communities represent large gaps in studies of urbanization and ecology (Hochkirch et al. 2021), which are only recently being addressed (Meyer et al. 2021; Lewthwaite et al. 2024). Given the general importance of fungal (Bahram and Netherway 2022; Seena et al. 2023) communities in shaping ecosystems, this is a gap which needs to be addressed to better understand the effects of urbanization on these taxa and ecosystems as a whole. Given the historically recent decline in global biodiversity (Pereira et al. 2024), addressing this gap is of particular urgency as the ecological impacts of anthropogenic stressors can often be tracked using the compositions of fungal communities (Zaghloul et al. 2020; Galitskaya et al. 2021). Prior evidence suggests that disturbances to soil from an increase in impervious coverage can lead to both a reduction in fungal richness, as well as a homogenization in their community compositions (Epp Schmidt et al. 2017).

Considering the presence of highly diverse, but understudied, fungal communities present in landscapes containing recently paved areas, we propose to investigate the following questions using publicly available data:

1. *Is the rise in impervious coverage reducing fungal richness in California?* Specifically, is there a significant difference in the richness of fungal communities found between areas with more or less impervious coverage?
2. *Is the rise of impervious coverage reducing fungal beta diversity dispersion in California?* Are the compositions of fungal communities significantly more similar to each other in areas with greater levels of impervious coverage?

## 2 Methods

### 2.1 Obtaining Impervious Coverage Maps

Our proxy measure for urbanization in this study is the percent impervious coverage as recorded by the National Land Cover Database (NLCD), which measures the fraction of land area within a 30 m square pixel covered by surfaces impervious to water flow such as concrete or asphalt (Homer et al. 2007; Homer 2015). We use this as a proxy for urbanization as it is associated with the use of impervious materials, which we expect to degrade the flow of nutrients through soil (Cambou et al. 2018; Lu et al. 2020), impacting fungal communities. We obtained maps of the percent impervious coverage for the continental United States, from the NLCD, for the years 2015 through 2024 from https://www.mrlc.gov/data. Each 30 m unit cell of these maps will hereafter be referred to as an imperviousness pixel. These maps were reprojected to the coordinate reference system (CRS) EPSG: 3857 and clipped to the boundaries of California using the GDAL function *gdalwarp* (Rouault 2023). The boundaries for California were obtained from the California Open Data Portal (https://data.ca.gov/dataset/ca‐geographic‐boundaries), and then reprojected to the CRS EPSG: 3857 using the GDAL function *ogr2ogr*.

### 2.2 Obtaining Additional Environmental Data

Additional environmental data were obtained from WorldClim (Fick and Hijmans 2017) and SoilGrids (Hengl et al. 2017). WorldClim is a global repository of long‐term climatic and elevation data at 30 arcsecond resolution, and was downloaded from https://worldclim.org/data/worldclim21.html. These map layers were reprojected to EPSG: 3857 using the function *project* within the R package *terra* (Hijmans 2023). These WorldClim layers were clipped to the boundaries of California. Slope and aspect were then calculated from elevation using the function *terrain* within *terra*.

SoilGrids contains global rasters, at 250 m resolution, of various soil conditions at various depths. Using Google Earth Engine (Gorelick et al. 2017), we downloaded the following environmental data for surface soils: bulk density, the clay content, organic carbon content, pH, sand content, and water content. These soil layers were clipped to the boundaries of California and resampled, using the *terra* function *resample*, to match the resolution of the WorldClim layers.

### 2.3 Obtaining and Filtering Fungal Data

Fungal occurrence data were obtained from the Global Biodiversity Information Facility (GBIF) using the following filters: all fungi within California for the years 2015 through 2024 (GBIF.org 2026). This initial data set contains 342,091 fungal occurrences. Of the initial 342,091 fungal occurrences 282,289 were derived from human observations, 1 from a living specimen, 12 from material citations, 55,090 from material samples, and 4699 from preserved specimens. These occurrences were then filtered to only retain those with species‐level assignments, reducing the data to 296,404 occurrences.

These fungal occurrences were then associated with ecological guilds, specifically trophic modes, using FUNGuild (Nguyen et al. 2016). The trophic mode assignments with a confidence ranking of ‘highly probable’ or ‘probable’ but which were ambiguous, that is those with multiple assigned categories such as ‘Pathotroph‐Saprotroph‐Symbiotroph’, were considered too ambiguous for meaningful ecological analyses and were not included in this analysis. Any occurrences with no assigned trophic mode, or without a ‘highly probable’ or ‘probable’ confidence ranking, were similarly removed from any analysis. This assignment process organized the remaining 179,892 occurrences into the following trophic modes: ‘symbiotroph’, ‘saprotroph’, and ‘pathotroph’ (Table 1).

**TABLE 1:** The number of fungal species occurrences, split by phylum and trophic mode, which are present in imperviousness pixels with at least 70%, 80%, and 90% sample coverage for a given year and trophic mode.

Sample coverage	phylum	Pathotroph	Saprotroph	Symbiotroph
70%	Ascomycota	167	705	3853
70%	Basidiomycota	88	5038	4447
70%	Chytridiomycota	0	5	0
70%	Glomeromycota	0	0	2
70%	Mucoromycota	0	137	0
80%	Ascomycota	163	677	3664
80%	Basidiomycota	79	4446	4257
80%	Chytridiomycota	0	5	0
80%	Glomeromycota	0	0	2
80%	Mucoromycota	0	137	0
90%	Ascomycota	132	639	2703
90%	Basidiomycota	45	2748	3778
90%	Chytridiomycota	0	5	0
90%	Glomeromycota	0	0	2
90%	Mucoromycota	0	137	0

To associate these fungal occurrences with an imperviousness pixel, we first split the occurrence data by year, then transformed them into a set of spatial data points in the CRS of EPSG:3857 using the functions *st_as_sf* and *st_transform* within the R package *sf* (Pebesma 2018). Each occurrence was then associated with a unique imperviousness pixel using the function *cellFromXY* within the R package *terra*, which allowed us to associate a unique identifier to each imperviousness pixel. The imperviousness pixel values were then associated with each occurrence using the *terra* function *extract*.

All fungal occurrences within a given year, trophic mode, and imperviousness pixel were, for the purposes of this study, treated as an assemblage. This approach does bin occurrences from a variety of collection events, seasons, sampling substrates, and so on, so these collections of occurrences are only proxies for true biological assemblages. However, the binning of occurrences from GBIF into unit cells from years' worth of data has been used before at various spatial scales (Troia and McManamay 2016; Lajeunesse and Fourcade 2023; El‐Gabbas 2026).

Given the use of opportunistically collected data, we accounted for variations in sampling effort by retaining our putative assemblages based on coverage thresholds. To test for the potential impact of choosing a particular coverage threshold, we performed our analyses at 70%, 80%, and 90% sample coverage thresholds. This was done by first estimating sample coverage using the function *DataInfo* within the R package *iNEXT* (Hsieh et al. 2016). This filtering, between no coverage threshold and one of 90%, reduced the number of putative fungal assemblages from 144,815 to 499, and the number of fungal occurrences used in this study to from 179,892 to 10,189 (Table 1).

### 2.4 Filtering and Extracting Environmental Data

To determine if any of the environmental variables are collinear, we first stacked all of the environmental layers and sampled their values, using the *terra* function *extract*, using the locations of the set of fungal occurrences filtered by sample coverage. Correlations between these extracted vectors were then calculated using the base R function *stats*, with ‘use’ set to the value of ‘complete.obs’. This correlation matrix was passed to the function *findCorrelation*, within the R package *caret* (Kuhn 2020), with a correlation threshold of 0.7. This identified environmental 8 layers (Table 2) which were collinear based on a Pearson correlation coefficient threshold of 0.7 (Barber et al. 2022). These environmental data were then extracted using the filtered set of fungal occurrences with the *terra* function *extract*.

**TABLE 2:** Environmental variables used in analyzing fungal diversity. Imperviousness has a resolution of 30 m; all other variables are at a resolution of 1045 m.

Variable	Description
Imperviousness	Fraction of 30 m pixel covered by impervious surfaces
Slope	Degrees slope
Aspect	Degrees aspect, counterclockwise from east
Bio2	Mean Diurnal Range (Mean of monthly range in temperatures)
Bio3	Isothermality, the ratio of the Mean Diurnal Range over the Annual Temperature Range
Bio13	Precipitation of Wettest Month
Bio15	Precipitation Seasonality (Coefficient of Variation)
Bio18	Precipitation of Warmest Quarter

### 2.5 Analyzing Occurrence Patterns

To test if the number of occurrences per assemblage and trophic mode varied significantly with either imperviousness or sample year, we first created a data frame containing the number of occurrences per assemblage, sample year, and imperviousness using the base R function *table*. This was done both before and after coverage‐based filtering. We then tested how the number of occurrences per assemblage varied with both sample year and imperviousness using the base R functions *lm* and *anova*. We calculated the skewness of our distribution of imperviousness values before and after filtering, using the value of three times the difference between the mean and median divided by the standard deviation.

To visualize the spatial distribution of these assemblages, and how they relate to imperviousness, we first summarized our imperviousness data by constructing an average imperviousness raster from our 2015–2024 data using the base R function *mean* on the stack of our imperviousness rasters. We plotted this averaged map layer using the *ggplot2* function *geom_raster*, with the location of fungal assemblages then plotted on top using the *ggplot2* function *geom_point*.

### 2.6 Alpha Diversity Analysis

As our occurrence data are opportunistically assembled, the sampling regime is effectively uneven across space and time. To be able to compare fungal richness between sampled locations over time, one may rarefy the occurrence data, although that would involve filtering out even more data. To better enable comparisons of alpha diversity taken between impervious coverage pixels and years, we estimated species richness using a method based on tracking the asymptotic behavior of richness versus sampling effort. This estimated species richness, our measure of alpha diversity, was calculated for each putative fungal assemblage using the function *iNEXT* within the R package *iNEXT* (Hsieh et al. 2016), with q (The Hill number) set to zero, the datatype set to abundance, and default settings otherwise. As our occurrence data were collected opportunistically, we have assumed an undercounting of rare species, so a q value of zero was selected to give equal weight to rare species in our estimations of richness. All of the estimated fungal species richness values, the imperviousness and environmental conditions they're associated with, and the year they were sampled were then aggregated into separate data sets for each trophic mode.

To visualize any potential trends between estimated fungal richness and log‐transformed imperviousness, we used the function *geom_point* within the R package *ggplot2*, along with a linear model overlaid using the *ggplot2* function *geom_smooth* (Wickham et al. 2016). To test if fungal richness within each trophic mode was significantly influenced by imperviousness, sample year, or other environmental conditions, we constructed a set of linear models of richness using all of those variables with the base R function *lm* and evaluated them with the base R function *summary*.

Given the large proportion of assemblages found in areas with no impervious coverage, we also conducted tests comparing fungal richness within each trophic mode between areas with and without any impervious cover. These tests were conducted using a one‐sided Wilcoxon rank‐sum test using the base R function ‘*wilcox.test*’. The distributions of fungal richness between areas with and without any impervious coverage were visualized using the ‘*ggplot2*’ function ‘*geom_boxplot*’.

### 2.7 Beta Diversity Analysis

Fungal occurrence data were filtered by sample coverage, split by trophic mode and sample year, and converted into a presence/absence matrix with the rows describing putative assemblages and columns the names of unique species. The beta diversity distance matrix for each fungal occurrence subset was then calculated using the function *vegdist*, within the R package *vegan* (Oksanen 2022), with the method selected as Jaccard and binary set to true.

To visualize the first two principal components of beta diversity, for each trophic mode, we ran the base R function *cmdscale*, with k set to 2, on our beta diversity distance matrices. These points were plotted, along with the percent variation attributed to the first two principal component axes, using the *ggplot2* function *geom_point*. Principal component plots were generated for assemblages from each trophic mode, and then colored by imperviousness.

Geographic distances between assemblages were determined using the function *distm*, within the R package *geosphere* (Hijmans 2017), with the distance function set to ‘distHaversine’. The locations of each assemblage were set as the centroids of the imperviousness pixels, as determined from the *terra* function *xyFromCell*. To find potential correlations between beta diversity and geographic distances, Mantel tests were run using the *vegan* function *mantel*, with 999 permutations and method set to ‘spearman’.

The amount and significance of the contributions of log‐transformed imperviousness, environmental conditions, geographic location, and year to variations in beta diversity for each trophic mode were calculated using the *vegan* function *adonis2* with 999 permutations and the method set to jaccard. Geographic location, specifically assemblage latitude and longitude, was included as Mantel tests did show significant correlations between beta diversity and geographic distance (Table S1).

The dispersion of each beta diversity distance matrix was calculated using the *vegan* function *betadisper* as the distance to the centroid of each group of assemblages grouped by trophic mode and year. To visualize potential trends in beta diversity dispersion against imperviousness we used the *ggplot2* function *geom_point*, with a linear fit overlaid using the base R function *lm*. Using the base R function *lm* and *summary* we then tested if fungal beta diversity dispersion, split by trophic mode, was significantly influenced by imperviousness, sample year, or other environmental conditions.

As our distribution of impervious coverage values was heavily skewed towards zero, we also conducted tests comparing fungal beta diversity dispersion for each trophic mode between areas with and without any impervious cover. These tests were conducted using a one‐sided Wilcoxon rank‐sum test using the base R function *wilcox.test*. Using the *ggplot2* function *geom_boxplot*, we then visualized the distributions of fungal beta diversity dispersion for each trophic mode between areas with and without any impervious coverage.

## 3 Results

### 3.1 Occurrence Patterns

Our initial set of fungal occurrences was heavily biased toward members of the phyla Ascomycota and Basidiomycota, of which most were of saprotrophic or symbiotrophic trophic modes (Table 1). Following our coverage‐based filtering, the number of occurrences was greatly reduced, but these same taxonomic and trophic biases remained (Table 1). We also found a strong positive trend between the number of occurrences and year both before and after filtering for sample coverage (Table S2).

Both prior to and following our coverage‐based filtering we found that the number of occurrences per assemblage often varied with both imperviousness and year, with year contributing more of the observed variation (Table S3). Across the totality of our data, we found a strong skew toward occurrences recorded in areas with low percent coverage (Figure 1). Prior to our coverage‐based filtering we found the skewness in our imperviousness distribution to be 1.64, while following filtering at a 90% coverage threshold it was 1.87. Given this skew toward low values we decided to analyze alpha and beta diversity against impervious coverage using two methods. The first method involves comparisons of alpha and beta diversity against log‐transformed imperviousness values as a continuous variable, calculated using the base R function *log1p*. The second method involves the comparisons of alpha and beta diversity for assemblages found in areas with and without any impervious coverage, treating imperviousness as a binary variable.

**FIGURE 1:**
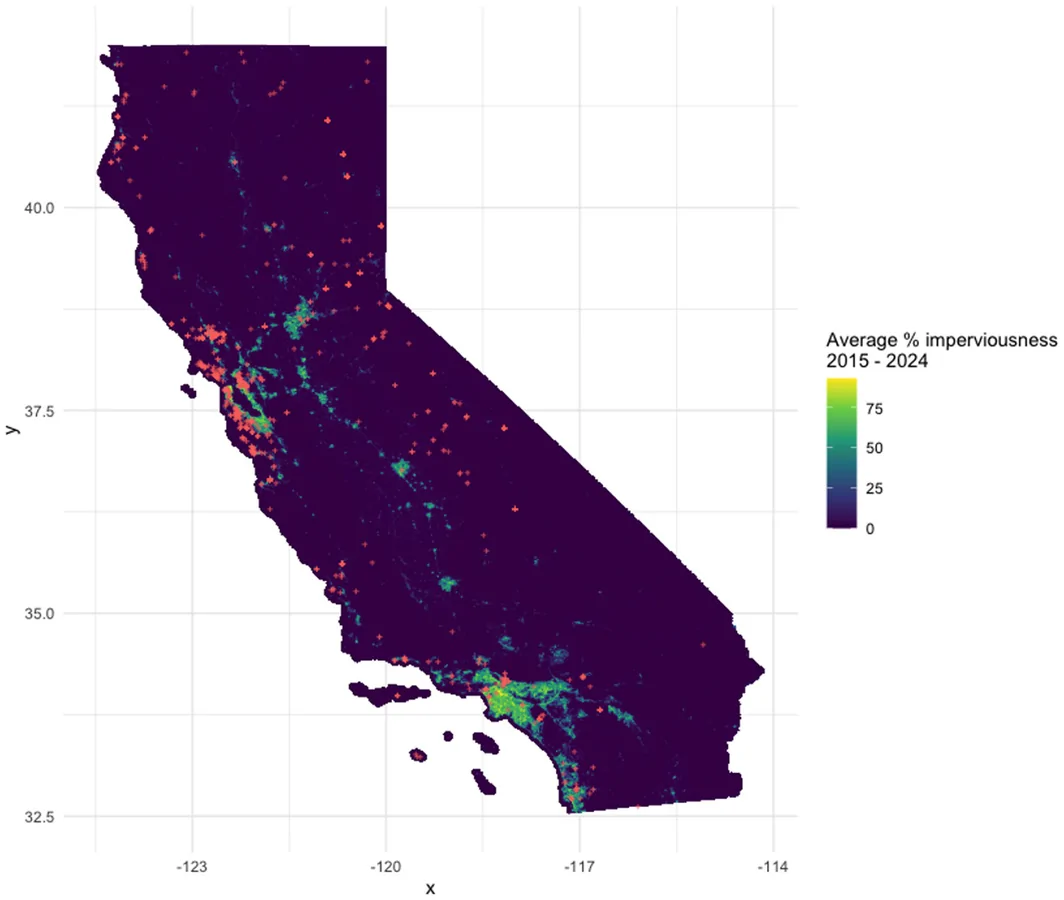
The average of imperviousness over 2015–2024, overlaid with assemblage locations marked in orange.

### 3.2 Alpha Diversity

We found evidence associating a decline in saprotrophic fungal richness with log‐transformed imperviousness (Table S4), although only for assemblages filtered at least at 90% coverage. Furthermore, most of this decline in richness appears to happen once there's any impermeable surface recorded in the immediate area (Figure S1). However, when comparing fungal assemblages found in areas with and without any impervious coverage, we did not observe a significant decline in richness (Figure S2, Table 3).

**TABLE 3:** W‐statistics and their significance scores, in parentheses, from Wilcoxon rank sum tests checking if richness is significantly lower for fungal assemblages sampled from areas with non‐zero impervious coverage.

Sample coverage	Pathotroph	Saprotroph	Symbiotroph
70%	129 (0.57)	95388.5 (0.69)	36960.5 (0.65)
80%	129 (0.66)	75,893 (0.32)	31,613 (0.73)
90%	8 (0.21)	9848.5 (0.17)	4208.5 (0.66)

The estimated richness of saprotrophic and symbiotrophic fungi was found to decline over time across coverage thresholds (Table S4). Saprotrophic fungal richness was also found to consistently decline with bio15 (the variability in precipitation) and increase with bio2 (the mean diurnal range in temperature) (Table S4). Saprotrophic fungal richness was found to decline with bio18 (the precipitation of the warmest month) but only for assemblages filtered at 70% or 80% coverage. Symbiotrohic fungal richness was found to consistently increase with bio13 (the precipitation of the wettest month).

### 3.3 Beta Diversity

For saprotrophic and symbiotrophic fungi, beta diversity was found to vary significantly with year and log‐transformed imperviousness (Figure S3, Table S5). Across sample coverage thresholds, we found variations in saprotrophic beta diversity to vary significantly with bio15, bio18, and bio2 (Table S5). Similarly, for symbiotrophic beta diversity, we found significant contributions to observed variations in beta diversity were associated with bio18 and bio3 (Table S5).

Dispersions in beta diversity, for saprotrophic and symbiotrophic assemblages, were consistently found to decline with log‐transformed imperviousness (Figure S4, Table S6). When comparing fungal assemblages found in areas with and without impervious coverage, we consistently found significant declines in beta diversity dispersion for both saprotrophic and symbiotrophic fungi (Figure S5, Table 4). Beta diversity dispersion consistently declines with time for symbiotrophic assemblages, and for saprotrophic assemblages filtered at 70% and 80% coverage (Table S6). For symbiotrophic fungi, beta diversity dispersion also appears to consistently decline with bio2 (Table S6).

**TABLE 4:** W‐statistics and their significance scores from Wilcoxon rank sum tests checking if beta diversity dispersion is significantly lower for fungal assemblages sampled from areas with non‐zero impervious coverage.

Sample coverage	Pathotroph	Saprotroph	Symbiotroph
70%	133.5 (0.63)	**65,897** (**1.95 10** ^**−14**^)	**21,674** (**7.54 × 10** ^**−15**^)
80%	136 (0.73)	**54,168** (**2.40 × 10** ^**−13**^)	**18,963** (**1.29 × 10** ^**−12**^)
90%	13 (0.61)	**6087** (**8.65 × 10** ^**−10**^)	**2196** (**1.83 × 10** ^**−7**^)

*Note:* Significant results are bolded.

## 4 Discussion

### 4.1 Occurrence Patterns

The fungal occurrences used in this analysis were, in effect, opportunistically assembled from a variety of sources in a single public database. Integrating various community science‐based observations can help greatly increase the volume of data for ecological analyses, although their geographic distribution tends to be biased toward readily accessible locations (Tiago et al. 2017). For the occurrence data used in this study, before and after filtering for assemblages with sufficient coverage, we found a persistent bias in sampling from areas with little to no impervious cover. This may, in part, reflect a previously observed bias in citizen science observations away from heavily urbanized areas and toward places that have unbuilt landcover but that are readily accessible by road (Mair and Ruete 2016).

The data used in this study are also quite recent, predominantly collected within a period of a few years, and unevenly sampled between years. Occurrences were also aggregated on an annual basis, obscuring potential seasonal variations in biodiversity patterns. While these data were aggregated on an annual basis to better enable calculations of biodiversity metrics in a given location, such aggregation may obscure variations in fungal observations between seasons or sampling substrates. For example, a number of fungal species are likelier to be detected in certain seasons coinciding with their fruiting events. Or, as we have seen in our data, there is a bias toward observations being made in unbuilt environments rather than any developed land. We also have a taxonomic bias in our data, with the majority of fungal occurrences being saprotrophic or symbiotrophic members of the phyla Ascomycota or Basidiomycota. Such taxonomic biases are not surprising as fungal citizen science is biased toward the detection of species which produce noticeable and readily distinguishable sporocarps (Haelewaters et al. 2024). However, as the volume of observations continues to grow it may become possible for there to be enough data to disentangle the effects of these observational biases on biodiversity estimates.

The volume of the fungal data used in this study were also found to increase on an annual basis, following the general trend toward an increase in community science observation for fungal diversity (Heilmann‐Clausen et al. 2019; Shumskaya et al. 2023; Haelewaters et al. 2024). However, we find that the size of the data set was large enough, even with the skew in the distribution of imperviousness values, that it was useful for testing our original hypotheses. What is interesting is that while prior studies of community science data have found geographic concentrations in and around populous cities, or accessible areas such as parks and near roads (Geldmann et al. 2016; Soroye et al. 2022), the fungal data in this study were heavily skewed toward areas with low levels of imperviousness.

### 4.2 Impervious Cover Is Associated With a Decline in Saprotrophic Fungal Richness

With fungal communities, we found richness to significantly decline for saprotrophic fungi in areas with more impervious cover, although only for assemblages filtered at a 90% coverage threshold. Similar declines in fungal diversity have been associated with urbanization elsewhere (Arnolds 1991; Abrego et al. 2020). This negative association between fungal diversity and impervious coverage may stem from a variety of factors, ranging from a reduction in the flow of nutrients across surface soils (Dighton et al. 2004) to habitat fragmentation (Merckx et al. 2018).

Using impervious surface coverage as a proxy for urbanization, our findings may reflect the impacts of urban soil eutrophication from urban runoff, which have been found to reduce fungal richness (Markkola et al. 1995; Tarvainen et al. 2003). An increase in surface runoff is also associated with an increase in impervious surface coverage, with rates of surface runoff found to double even when only 10%–20% of an area is rendered impervious (Paul and Meyer 2001). This leads to a significant reduction, by up to 50%, in groundwater recharge in urbanized areas (Hough 2002), which would also be expected to reduce resources to support greater fungal diversity.

A rise in impervious coverage is also associated with habitat fragmentation in urban environments, which often reduces soil habitats for fungi below a viable threshold to maintain diversity within a patch, thus leading to broader declines in urban areas (Drinnan 2005). These declines in fungal richness have been connected with disturbances to plant communities and their fungal symbionts due to land‐use changes associated with urbanization (Epp Schmidt et al. 2017; Delgado‐Baquerizo et al. 2021). Changes in air temperature and rainfall, both influenced by the urban heat‐island effect, have been found to be important drivers of fungal taxonomic richness (Delgado‐Baquerizo et al. 2021; Gómez‐Hernández et al. 2021).

### 4.3 Impervious Cover Is Associated With Reduced Saprotrophic and Symbiotrophic Beta Diversity Dispersion

We found imperviousness to be significantly associated with a reduction in beta diversity dispersion for saprotrophic and symbiotrophic assemblages, implying that urbanization is significantly associated with the homogenization of these fungal communities. We also find that, unlike other factors such as year, location, or other environmental conditions, imperviousness was a strong and consistent factor associated with this reduction in beta diversity dispersion for these fungal assemblages. While we did not find any significant trends in pathotrophic fungal beta diversity dispersion in relation to impervious coverage, it is unclear if this reflects a true ecological signal or poor statistics related to the relatively small number of recorded occurrences.

We propose that this reduction in beta diversity dispersion could at least in part reflect ecological filtering, due to the stresses associated with urbanization such as disturbances to the soil from mechanical and chemical alterations. This result may reflect prior evidence of a significant role for urbanization in shaping beta diversity for fungal communities (Epp Schmidt et al. 2017; Rusterholz and Baur 2023). The observed decrease in beta diversity dispersion associated with imperviousness may relate to prior land conversion associated with urbanization, which has been found to disrupt the complex structure of mycorrhizal networks (Authier et al. 2022). Land conversion can influence both local climate, as well as types of soil available, which can result in changes in beta diversity for fungal communities (Epp Schmidt et al. 2017; Delgado‐Baquerizo et al. 2021; Mikryukov et al. 2023). There is also prior evidence of enhanced heavy metal runoff from large impervious areas, such as road networks, driving changes in fungal communities (Luo et al. 2025).

The observed reductions in fungal beta diversity dispersion may also stem from alterations in their associated plant communities due to an increase in impervious cover in an area, such as losses to native plant diversity. The structure of fungal communities is strongly associated with plant communities, and while urbanization is associated with a decline in native plant coverage there is often an associated rise in the spread of non‐native plants (Pyšek 1998; Rapoport 1993; Tait et al. 2005). This process can filter fungal populations, leading to a more distinctly urban fungal community type (Newbound et al. 2010; Whitehead et al. 2022; Scholier et al. 2023). Such urban‐associated ecological filtering of fungal communities has been observed elsewhere (Aguilar‐Trigueros et al. 2025). Urbanization has also led to a great increase in the transport of soil. Soil transportation can result in both the transport of introduced species, as well as the homogenization of soil communities, thereby reducing beta diversity (La Sorte et al. 2014; Blouin et al. 2019; Wang et al. 2021). Urbanization in general has been found to both homogenize habitat, and as a result, the structure of biological communities such as fungi (Groffman et al. 2017; Kuhn and Klotz 2006; Pearse et al. 2018).

### 4.4 Synthesis and Future Directions

There is a growing need to monitor the ecological effects in an increasingly urbanized world. Of particular importance are the impacts of urbanization on the structure of fungal communities, which underpin the functioning of many ecosystems. While fungi are relatively underrepresented in citizen science compared to a number of other taxonomic groups (Cerrejón et al. 2025), on the geographic scale of California there is now enough data to assess environmental trends influencing fungal biodiversity. This trend, toward ever greater amounts of fungal diversity data, is set to continue or even grow with the rise in the use of environmental DNA (Heine et al. 2021; Policelli 2024). With eDNA, a wide variety of fungal species can be better resolved than by morphology alone (Tedersoo et al. 2025), or a number of microscopic fungal species can be identified from soil samples which would otherwise escape human observation (Frøslev et al. 2019). The great promise for future monitoring and management efforts is that the fungal datasets are expected to continue to expand rapidly in both volume and taxonomic resolution (Saki and Yadav 2025). The evidence so far points to the significant impact of impervious coverage on fungal diversity patterns, which is only set to be more reliable to study in more or even smaller areas for precision ecological monitoring.

## Author Contributions

**Ariel Levi Simons:** conceptualization (lead), formal analysis (lead), investigation (lead), methodology (lead), software (lead), supervision (lead), validation (lead), visualization (lead), writing – original draft (equal), writing – review and editing (equal). **Christopher Timlin‐Broussard:** writing – original draft (equal), writing – review and editing (equal). **Avicka Willis:** writing – original draft (equal), writing – review and editing (equal). **Jerelyn Lee:** writing – original draft (equal), writing – review and editing (equal).

## Conflicts of Interest

The authors declare no conflicts of interest.

## Supporting information


**Figure S1:** Estimated fungal richness versus the log of impervious coverage for assemblages filtered at 70% (A), 80% (B), and 90% (C) coverage.
**Figure S2:** Estimated fungal richness for assemblages with and without any impervious coverage for assemblages filtered at 70% (A), 80% (B), and 90% (C) coverage.
**Figure S3:** PCoA plot of Jaccard beta diversity of assemblages split by trophic mode and colored by imperviousness.
**Figure S4:** Beta diversity dispersion versus the log of impervious coverage for fungal assemblages filtered at 70% (A–C), 80% (D–F), and 90% (G–I) coverage.
**Figure S5:** Beta diversity dispersion for assemblages with and without any impervious coverage for fungal assemblages filtered at 70% (A–C), 80% (D–F), and 90% (G–I) coverage.
**Table S1:** Mantel test results between the Jaccard dissimilarity and geographic separation distances of fungal assemblages split by trophic mode.
**Table S2:** Spearman correlations between the number of occurrences per fungal assemblage and year for different sample coverage thresholds.
**Table S3:** ANOVA results on variations in the number of occurrences per assemblage, split by trophic mode, against both year and imperviousness for assemblages filtered at different coverage thresholds. Significant results are bolded.
**Table S4:** Summary of linear models of fungal richness, grouped by trophic mode, against sample year, imperviousness, and other environmental variables. Samples are filtered at various coverage thresholds. Significant results are bolded.
**Table S5:** PERMANOVA results, using 999 permutations, on the significance of various factors to observed variations in Jaccard dissimilarity for groups of assemblages split by trophic modes and filtered at different coverage thresholds. Significant results are bolded.
**Table S6:** Summary of linear models of fungal beta diversity dispersion, grouped by trophic mode, against sample year, imperviousness, location, and other environmental variables. Samples are filtered at various coverage thresholds. Significant results are bolded.

## Data Availability

All of the source data and code used in this analysis is available via the following archive: https://doi.org/10.5061/dryad.ttdz08mdm.
