# Supplementary material for: Evidence for Reductions in Fungal Richness and Beta Diversity Dispersion Associated With Impervious Surfaces in California
Source: Ecol Evol. 2026 Sep 29;16(10):e74448. doi: 10.1002/ece3.74448 (PMC13623452; doi:10.1002/ece3.74448)
Supplement: Supplementary file 1 [file ECE3-16-e74448-s001.docx]

**Supplementary figures**

Figure S1 - Estimated fungal richness versus the log of impervious coverage for assemblages filtered at 70% (A), 80% (B), and 90% (C) coverage.

Figure S2 - Estimated fungal richness for assemblages with and without any impervious coverage for assemblages filtered at 70% (A), 80% (B), and 90% (C) coverage.

Figure S3 - PCoA plot of Jaccard beta diversity of assemblages split by trophic mode and colored by imperviousness.

Figure S4 - Beta diversity dispersion versus the log of impervious coverage for fungal assemblages filtered at 70% (A - C), 80% (D - F), and 90% (G - I) coverage.

Figure S5 - Beta diversity dispersion for assemblages with and without any impervious coverage for fungal assemblages filtered at 70% (A - C), 80% (D - F), and 90% (G - I) coverage.

**Supplementary tables**

Table S1 - Mantel test results between the Jaccard dissimilarity and geographic separation distances of fungal assemblages split by trophic mode.

| Coverage threshold | Trophic mode | 𝟈 | p |
| --- | --- | --- | --- |
| 70% | Pathotroph | 0.42 | 10^-3^ |
| 70% | Saprotroph | 0.07 | 10^-3^ |
| 70% | Symbiotroph | 0.11 | 10^-3^ |
| 80% | Pathotroph | 0.44 | 10^-3^ |
| 80% | Saprotroph | 0.07 | 10^-3^ |
| 80% | Symbiotroph | 0.11 | 10^-3^ |
| 90% | Pathotroph | 0.54 | 5 x 10^-3^ |
| 90% | Saprotroph | 0.10 | 10^-3^ |
| 90% | Symbiotroph | 0.10 | 10^-3^ |

Table S2 - Spearman correlations between the number of occurrences per fungal assemblage and year for different sample coverage thresholds.

| Sample coverage threshold | 𝟈 | p |
| --- | --- | --- |
| 0% | 0.96 | < 2.2 x 10^-16^ |
| 70% | 0.87 | 2.68 x 10^-3^ |
| 80% | 0.85 | 1.79 x 10^-3^ |
| 90% | 0.71 | 0.03 |

Table S3 – ANOVA results on variations in the number of occurrences per assemblage, split by trophic mode, against both year and imperviousness for assemblages filtered at different coverage thresholds. Significant results are bolded.

| Coverage threshold | Trophic mode | Number of assemblages | F  (year) | p  (year) | F  (imperviousness) | p  (imperviousness) |
| --- | --- | --- | --- | --- | --- | --- |
| 0% | Pathotroph | 10,462 | **9.13 x 10^2^** | **< 2.2 x 10^-16^** | **23.3** | **1.40 x 10^-6^** |
| 0% | Saprotroph | 82,745 | **9.65 x 10^3^** | **< 2.2 x 10^-16^** | **6.43 x 10^2^** | **< 2.2 x 10^-16^** |
| 0% | Symbiotroph | 51,608 | **1.69 x 10^4^** | **< 2.2 x 10^-16^** | **6.28 x 10^2^** | **< 2.2 x 10^-16^** |
| 70% | Pathotroph | 35 | **8.38** | **6.79 x 10^-3^** | 1.19 x 10^-2^ | 0.91 |
| 70% | Saprotroph | 849 | **44.3** | **5.13 x 10^-11^** | **4.24** | **0.04** |
| 70% | Symbiotroph | 594 | **21.1** | **5.46 x 10^-6^** | 1.69 | 0.19 |
| 80% | Pathotroph | 34 | **7.91** | **8.46 x 10^-3^** | 1.27 x 10^-2^ | 0.91 |
| 80% | Saprotroph | 771 | **44.3** | **5.26 x 10^-11^** | **4.48** | **0.03** |
| 80% | Symbiotroph | 545 | **20.8** | **6.34 x 10^-6^** | 1.74 | 0.19 |
| 90% | Pathotroph | 14 | 3.14 | 0.10 | 1.53 x 10^-2^ | 0.90 |
| 90% | Saprotroph | 285 | **57.3** | **5.33 x 10^-13^** | **7.19** | **7.78 x 10^-3^** |
| 90% | Symbiotroph | 200 | **22.6** | **3.83 x 10^-6^** | 2.74 | 0.10 |

Table S4 - Summary of linear models of fungal richness, grouped by trophic mode, against sample year, imperviousness, and other environmental variables. Samples are filtered at various coverage thresholds. Significant results are bolded.

| Coverage threshold | Factor | Pathotroph  t-value (p) | Saprotroph  t-value (p) | Symbiotroph  t-value (p) |
| --- | --- | --- | --- | --- |
| 70% | year | 1.29 (0.21) | **-2.82 (4.92 x 10^-3^)** | **-2.14 (3.31 x 10^-2^)** |
| 70% | Ln(1 + imperviousness) | -1.30 (0.21) | 0.71 (0.48) | 0.79 (0.43) |
| 70% | slope | -5.7 x 10^-2^ (0.96) | -0.24 (0.81) | 1.37 (0.17) |
| 70% | aspect | 0.16 (0.87) | -0.40 (0.69) | -1.31 (0.19) |
| 70% | bio13 | -0.57 (0.58) | 1.07 (0.29) | **2.63 (8.85 x 10^-3^)** |
| 70% | bio15 | 0.21 (0.84) | **-3.24 (1.25 x 10^-3^)** | -0.46 (0.65) |
| 70% | bio18 | 0.20 (0.84) | **-2.07 (3.84 x 10^-2^)** | 0.53 (0.60) |
| 70% | bio2 | -6.9 x 10^-2^ (0.95) | **4.19 (3.09 x 10^-5^)** | **2.40 (1.66 x 10^-2^)** |
| 70% | bio3 | 1.06 (0.30) | 1.68 (9.36 x 10^-2^) | 0.32 (0.75) |
| 80% | year | **-2.61 (1.54 x 10^-2^)** | **-4.65 (3.85 x 10^-6^)** | **-2.24 (2.52 x 10^-2^)** |
| 80% | Ln(1 + imperviousness) | 1.36 (0.19) | 0.81 (0.42) | 0.69 (0.49) |
| 80% | slope | 1.59 (0.12) | -0.25 (0.80) | 1.66 (9.68 x 10^-2^) |
| 80% | aspect | 0.72 (0.48) | -0.62 (0.54) | -1.11 (0.27) |
| 80% | bio13 | **-2.37 (2.61 x 10^-2^)** | 0.88 (0.38) | **2.12 (3.44 x 10^-2^)** |
| 80% | bio15 | 1.36 (0.18) | **-4.00 (7.05 x 10^-5^)** | -0.74 (0.46) |
| 80% | bio18 | 1.68 (0.11) | **-2.05 (4.06 x 10^-2^)** | 0.37 (0.71) |
| 80% | bio2 | -0.34 (0.74) | **4.53 (6.76 x 10^-6^)** | 1.90 (5.81 x 10^-2^) |
| 80% | bio3 | -5.52 x 10^-2^ (0.96) | 0.49 (0.62) | 0.52 (0.60) |
| 90% | year | 2.38 (7.71 x 10^-2^) | **-6.26 (1.43 x 10^-9^)** | **-4.70 (4.94 x 10^-6^)** |
| 90% | Ln(1 + imperviousness) | 1.45 (0.22) | **-2.21 (2.78 x 10^-2^)** | -0.77 (0.45) |
| 90% | slope | 1.60 (0.19) | -0.65 (0.51) | -0.91 (0.36) |
| 90% | aspect | 0.61 (0.58) | -0.40 (0.69) | 0.17 (0.86) |
| 90% | bio13 | -2.25 (8.75 x 10^-2^) | -0.31 (0.75) | **2.10 (3.68 x 10^-2^)** |
| 90% | bio15 | 2.19 (9.33 x 10^-2^) | **-3.07 (2.37 x 10^-3^)** | -1.64 (0.10) |
| 90% | bio18 | 1.83 (0.14) | -1.30 (0.20) | -1.37 (0.17) |
| 90% | bio2 | -0.90 (0.42) | **2.78 (2.35 x 10^-2^)** | 1.02 (0.31) |
| 90% | bio3 | -1.22 (0.29) | 0.36 (0.72) | -0.81 (0.42) |

Table S5 - PERMANOVA results, using 999 permutations, on the significance of various factors to observed variations in Jaccard dissimilarity for groups of assemblages split by trophic modes and filtered at different coverage thresholds. Significant results are bolded.

| Coverage threshold | Factor | Pathotroph | | | Saprotroph | | | Symbiotroph | | |
| --- | --- | --- | --- | --- | --- | --- | --- | --- | --- | --- |
|  |  | F | n | p | F | n | p | F | n | p |
| 70% | Longitude | 1.35 | 24 | 0.14 | **1.65** | **855** | **1.3 x 10^-2^** | **2.77** | **586** | **10^-3^** |
| 70% | Latitude | 1.23 | 24 | 0.21 | **1.39** | **855** | **4.7 x 10^-2^** | **2.31** | **586** | **10^-3^** |
| 70% | year | 1.41 | 24 | 0.13 | **3.87** | **855** | **10^-3^** | **2.94** | **586** | **10^-3^** |
| 70% | Ln(1 + imperviousness) | 1.40 | 24 | 0.13 | **9.78** | **855** | **10^-3^** | **3.25** | **586** | **10^-3^** |
| 70% | slope | 0.99 | 24 | 0.40 | 1.11 | 855 | 0.27 | 1.02 | 586 | 0.40 |
| 70% | aspect | 1.11 | 24 | 0.29 | 1.10 | 855 | 0.27 | 1.09 | 586 | 0.23 |
| 70% | bio13 | 1.16 | 24 | 0.23 | **2.25** | **855** | **10^-3^** | **2.60** | **586** | **10^-3^** |
| 70% | bio15 | 1.51 | 24 | 0.08 | **2.49** | **855** | **10^-3^** | **1.68** | **586** | **10^-3^** |
| 70% | bio18 | 1.27 | 24 | 0.16 | **1.77** | **855** | **5 x 10^-3^** | **1.72** | **586** | **10^-3^** |
| 70% | bio2 | **1.72** | **24** | **4.7 x 10^-2^** | **2.82** | **855** | **10^-3^** | **2.05** | **586** | **10^-3^** |
| 70% | bio3 | **2.03** | **24** | **2.3 x 10^-2^** | **1.44** | **855** | **3.2 x 10^-2^** | **2.61** | **586** | **10^-3^** |
| 80% | Longitude | 1.39 | 23 | 0.13 | **1.57** | **775** | **1.5 x 10^-2^** | **2.54** | **537** | **10^-3^** |
| 80% | Latitude | 1.27 | 23 | 0.20 | 1.32 | 775 | 6.5 x 10^-2^ | **2.22** | **537** | **10^-3^** |
| 80% | year | 1.44 | 23 | 0.11 | **3.67** | **775** | **10^-3^** | **2.79** | **537** | **10^-3^** |
| 80% | Ln(1 + imperviousness) | 1.47 | 23 | 0.10 | **9.57** | **775** | **10^-3^** | **2.56** | **537** | **10^-3^** |
| 80% | slope | 1.00 | 23 | 0.44 | 1.06 | 775 | 0.34 | 0.98 | 537 | 0.54 |
| 80% | aspect | 1.18 | 23 | 0.23 | 1.06 | 775 | 0.34 | 0.98 | 537 | 0.54 |
| 80% | bio13 | 1.23 | 23 | 0.21 | **2.13** | **775** | **10^-3^** | **2.57** | **537** | **10^-3^** |
| 80% | bio15 | 1.46 | 23 | 0.10 | **2.33** | **775** | **10^-3^** | **1.81** | **537** | **10^-3^** |
| 80% | bio18 | 1.31 | 23 | 0.14 | **1.77** | **775** | **7 x 10^-3^** | **1.73** | **537** | **10^-3^** |
| 80% | bio2 | **1.75** | **23** | **4.3 x 10^-2^** | **2.58** | **775** | **10^-3^** | **1.91** | **537** | **10^-3^** |
| 80% | bio3 | **2.03** | **23** | **2.3 x 10^-2^** | 1.33 | 775 | 7.3 x 10^-2^ | **2.48** | **537** | **10^-3^** |
| 90% | Longitude | 3.81 | 14 | 0.06 | 1.26 | 288 | 0.10 | **1.48** | **202** | **4 x 10^-3^** |
| 90% | Latitude | 2.49 | 14 | 0.10 | 1.25 | 288 | 0.10 | **1.45** | **202** | **4 x 10^-3^** |
| 90% | year | 3.33 | 14 | 0.07 | **2.45** | **288** | **10^-3^** | **1.62** | **202** | **3 x 10^-3^** |
| 90% | Ln(1 + imperviousness) | 2.47 | 14 | 0.11 | **5.17** | **288** | **10^-3^** | **1.84** | **202** | **10^-3^** |
| 90% | slope | 0.98 | 14 | 0.52 | 1.29 | 288 | 0.11 | 0.98 | 202 | 0.52 |
| 90% | aspect | **6.77** | **14** | **0.01** | **1.38** | **288** | **4.5 x 10^-2^** | 1.12 | 202 | 0.16 |
| 90% | bio13 | **10.54** | **14** | **7.0 x 10^-3^** | 1.26 | 288 | 0.11 | 1.23 | 202 | 6.2 x 10^-2^ |
| 90% | bio15 | **13.06** | **14** | **5.0 x 10^-3^** | **1.40** | **288** | **4.3 x 10^-2^** | 1.19 | 202 | 8.5 x 10^-2^ |
| 90% | bio18 | **12.58** | **14** | **6.0 x 10^-3^** | **1.49** | **288** | **3.9 x 10^-2^** | **1.42** | **202** | **8 x 10^-3^** |
| 90% | bio2 | 2.57 | 14 | 8.7 x 10^-2^ | **2.79** | **288** | **10^-3^** | 1.23 | 202 | 5.5 x 10^-2^ |
| 90% | bio3 | 2.15 | 14 | 0.12 | 1.19 | 288 | 0.16 | **1.66** | **202** | **10^-3^** |

Table S6 - Summary of linear models of fungal beta diversity dispersion, grouped by trophic mode, against sample year, imperviousness, location, and other environmental variables. Samples are filtered at various coverage thresholds. Significant results are bolded.

| Coverage threshold | Factor | Pathotroph  t-value (p) | Saprotroph  t-value (p) | Symbiotroph  t-value (p) |
| --- | --- | --- | --- | --- |
| 70% | Longitude | 0.64 (0.53) | 1.33 (0.18) | **4.15 (3.76 x 10^-5^)** |
| 70% | Latitude | 0.12 (0.91) | 0.36 (0.72) | **2.51 (1.23 x 10^-2^)** |
| 70% | year | -0.57 (0.57) | **-4.25 (2.40 x 10^-5^)** | **-5.44 (7.71 x 10^-8^)** |
| 70% | Ln(1 + imperviousness) | 1.08 (0.29) | **-2.66 (8.06 x 10^-3^)** | **-5.82 (9.79 x 10^-9^)** |
| 70% | slope | 0.24 (0.81) | -1.31 (0.19) | 1.41 (0.16) |
| 70% | aspect | 0.09 (0.93) | -0.87 (0.39) | 0.25 (0.80) |
| 70% | bio13 | -0.50 (0.63) | **3.70 (2.29 x 10^-4^)** | 1.55 (0.12) |
| 70% | bio15 | -1.19 (0.24) | **-2.41 (1.61 x 10^-2^)** | -0.77 (0.44) |
| 70% | bio18 | 0.08 (0.94) | 1.09 (0.28) | 1.84 (6.70 x 10^-2^) |
| 70% | bio2 | -1.89 (0.07) | 0.09 (0.93) | **-3.37 (8.15 x 10^-4^)** |
| 70% | bio3 | 1.95 (0.06) | 0.49 (0.63) | **1.99 (4.72 x 10^-2^)** |
| 80% | Longitude | 0.63 (0.53) | 1.50 (0.13) | **4.28 (2.18 x 10^-5^)** |
| 80% | Latitude | 0.20 (0.84) | 0.85 (0.40) | **2.43 (1.53 x 10^-2^)** |
| 80% | year | -0.56 (0.58) | **-3.87 (1.18 x 10^-4^)** | **-4.98 (8.48 x 10^-7^)** |
| 80% | Ln(1 + imperviousness) | 1.20 (0.24) | **-2.57 (1.02 x 10^-2^)** | **-4.71 (3.20 x 10^-6^)** |
| 80% | slope | 0.44 (0.67) | -1.17 (0.24) | -1.39 (0.16) |
| 80% | aspect | 0.16 (0.88) | -1.01 (0.31) | -0.17 (0.87) |
| 80% | bio13 | -0.69 (0.50) | **3.06 (2.28 x 10^-3^)** | **2.03 (4.31 x 10^-2^)** |
| 80% | bio15 | -0.92 (0.37) | -1.69 (9.10 x 10^-2^) | -1.12 (0.26) |
| 80% | bio18 | 0.26 (0.80) | 1.24 (0.22) | 1.55 (0.12) |
| 80% | bio2 | -1.83 (8.03 x 10^-2^) | 0.05 (0.96) | **-2.93 (3.55 x 10^-3^)** |
| 80% | bio3 | 1.79 (8.70 x 10^-2^) | 0.23 (0.82) | **2.57 (1.05 x 10^-2^)** |
| 90% | Longitude | 3.24 (8.33 x 10^-2^) | 1.30 (0.19) | 1.05 (0.29) |
| 90% | Latitude | 0.73 (0.54) | 1.29 (0.20) | 0.68 (0.50) |
| 90% | year | -2.49 (0.13) | -0.53 (0.59) | **-2.92 (3.97 x 10^-3^)** |
| 90% | Ln(1 + imperviousness) | -1.16 (0.36) | **-2.24 (2.59 x 10^-2^)** | **-4.11 (5.89 x 10^-5^)** |
| 90% | slope | 1.92 (0.20) | -1.51 (0.13) | -1.38 (0.17) |
| 90% | aspect | **-4.33 (4.94 x 10^-2^)** | -0.86 (0.39) | -0.99 (0.33) |
| 90% | bio13 | **6.32 (2.42 x 10^-2^)** | 1.37 (0.17) | 0.25 (0.81) |
| 90% | bio15 | **-6.89 (2.04 x 10^-2^)** | 0.07 (0.94) | -0.73 (0.46) |
| 90% | bio18 | **-7.05 (1.95 x 10^-2^)** | 1.55 (0.12) | 1.01 (0.31) |
| 90% | bio2 | -0.43 (0.71) | **2.09 (3.74 x 10^-2^)** | **-2.25 (2.58 x 10^-2^)** |
| 90% | bio3 | 2.53 (0.13) | -0.23 (0.82) | 0.50 (0.62) |
